# Do postoperative complications correlate to chronic pain following inguinal hernia repair? A prospective cohort study from the Swedish Hernia Register

**DOI:** 10.1007/s10029-021-02545-y

**Published:** 2021-12-11

**Authors:** A. Olsson, G. Sandblom, U. Franneby, A. Sondén, U. Gunnarsson, U. Dahlstrand

**Affiliations:** 1 Department of Clinical Science and Education Södersjukhuset, Karolinska Institutet, Stockholm, Sweden; 2 Department of Clinical Science, Intervention and Technology, Karolinska Institutet, Stockholm, Sweden; 3 Department of Surgical and Perioperative Sciences, Surgery, Umeå University, Umeå, Sweden; 4 Department of Surgery, Södersjukhuset, Stockholm, Sweden

**Keywords:** Inguinal hernia, Groin hernia, Chronic pain, Postoperative complications

## Abstract

**Purpose:**

To analyse if postoperative complications constitute a predictor for the risk of developing long-term groin pain.

**Methods:**

Population-based prospective cohort study of 30,659 patients operated for inguinal hernia 2015–2017 included in the Swedish Hernia Register. Registered post-operative complications were categorised into hematomas, surgical site infections, seromas, urinary tract complications, and acute post-operative pain. A questionnaire enquiring about groin pain was distributed to all patients 1 year after surgery. Multivariable logistic regression analysis was used to find any association between postoperative complications and reported level of pain 1 year after surgery.

**Results:**

The response rate was 64.5%. In total 19,773 eligible participants responded to the questionnaire, whereof 73.4% had undergone open anterior mesh repair and 26.6% had undergone endo-laparoscopic mesh repair.

Registered postoperative complications were: 750 hematomas (2.3%), 516 surgical site infections (1.6%), 395 seromas (1.2%), 1216 urinary tract complications (3.7%), and 520 hernia repairs with acute post-operative pain (1.6%).

Among patients who had undergone open anterior mesh repair, an association between persistent pain and hematomas (OR 2.03, CI 1.30–3.18), surgical site infections (OR 2.18, CI 1.27–3.73) and acute post-operative pain (OR 7.46, CI 4.02–13.87) was seen. Analysis of patients with endo-laparoscopic repair showed an association between persistent pain and acute post-operative pain (OR 9.35, CI 3.18–27.48).

**Conclusion:**

Acute postoperative pain was a strong predictor for persistent pain following both open anterior and endo-laparoscopic hernia repair. Surgical site infection and hematoma were predictors for persistent pain following open anterior hernia repair, although the rate of reported postoperative complications was low.

## Introduction

Approximately 20 million hernia repairs are performed annually worldwide [1]. The lifetime risk for groin hernia surgery is reported to be 27% in men and 3% in women [2]. Using modern surgical techniques, the main adverse outcome after inguinal hernia surgery is Chronic Postoperative Inguinal Pain (CPIP)[3]. Chronic pain is defined as pain lasting more than 3 months [4].

CPIP is associated with disability, dissatisfaction, impaired productivity, and decreased quality of life [3]. Studies have reported the prevalence of CPIP to 20–30%, while severe CPIP, affecting daily activities, has been reported to occur in 6–10% at long-term follow-ups [5, 6]. The proportion of patients with persistent pain decreases with time but persists more than 7 years after surgery in 14% of the cases [7].

Efforts to identify and reduce risks for persisting pain is crucial. Risk factors for chronic pain after inguinal hernia repair can be divided in patient-related risk factors, such as young age, female sex, preoperative pain, significant pain in other locations, and specific genotypes [8–11]; and surgery-related risk factors, such as open hernia repair, also including the proposed risk factors of nerve handling, choice of mesh material and method for fixation [12, 13].

Previous studies from our research group have suggested that postoperative complications are also risk factors for CPIP [5]. Patients with a postoperative complication registered in the Swedish Hernia Register (SHR) were more likely to report some degree of persisting pain in the operated groin, when responding to the Inguinal Pain Questionnaire (IPQ) 2–3 years after hernia repair [5]. In an 8-year follow-up of another cohort of hernia surgery patients, those who had reported severe pain in the early post-operative period were more likely to report pain in the operated groin or testicular pain at the follow-up [14]. Patients who had reported urinary tract complications were also more prone to ipsilateral testicular pain 8 years after hernia surgery [14]. In this study, answers to a self-report questionnaire administered 1 month after surgery regarding postoperative complications were used to define whether a specific complication had occurred or not [14, 15].

Since patient satisfaction has become an important measure of successful outcome, a Patient Report Outcome Measure (PROM) questionnaire has been developed by the SHR. The PROM was automatically sent 1 year after surgery to all registered patients that had undergone hernia repair [16]. The SHR has also updated the register form with more detailed registration of postoperative complications, providing valuable information on health-service assessed complications [16].

In this study we investigated the impact of specific postoperative complications on CPIP in a population-based material, using data from the extended SHR form and corresponding PROM answers.

The hypothesis in this study was that some postoperative complications increase the risk for chronic pain after inguinal hernia repair. The aim of this study was to clarify the association between specific postoperative complications and CPIP in the operated groin with the primary endpoint patient-reported persistent pain 1 year after surgery.

## Materials and methods

This study was designed as a population-based prospective cohort study. Collection of data was performed in accordance with the RECORD statement extended from the STROBE statement for observational studies [17]. Data were obtained from the Swedish Hernia Register (SHR), a national register of groin hernia repairs in adults. The SHR was established in 1992 and over the last two decades coverage of all repairs performed in Sweden has been > 95% [16]. Data are recorded prospectively after each procedure. Information regarding patient characteristics, hernia characteristics, method of repair and procedural details is registered. Postoperative complications within 30 days from the procedure are recorded. One year after surgery, the SHR sends a PROM questionnaire to all patients by mail enquiring about persisting groin pain, persisting groin complaints, the result of the operation, and their global experience of the surgical procedure. No reminders are sent, due to the large cohort and the logistic and financial implications of such administration.

Patients who had undergone inguinal hernia repair between 27th August 2015 and 31st August 2017 and were registered in the SHR were eligible for inclusion. The starting date of the study was chosen since that was when the SHR began more detailed registration of postoperative complications.

Inclusion criteria were registered hernia repair and a completed PROM. Exclusion criteria were hernia repair techniques other than open anterior mesh repair and endo-laparoscopic repair with preperitoneal mesh placement. “Other repairs” were excluded due to the heterogeneity of that category. Open and endo-laparoscopic repairs were analysed as two separate groups.

The primary outcome was reported level of pain during the past week reported in the PROM questionnaire. The PROM included a seven-grade ordinal scale rating of persistent pain in the operated groin, derived from the former Inguinal Pain Questionnaire (IPQ) [18, 19]. Grading of intensity of pain was: 1—no pain; 2—pain can be ignored; 3—pain cannot be ignored, but it does not affect everyday activities; 4—pain cannot be ignored, and it affects everyday activities; 5—pain prevents most activities; 6—pain necessitates bed rest; and 7—pain requires immediate medical attention.

Definitions of the postoperative complications included in the SHR during the study period are listed in Table 1.

**Table 1:** Postoperative complications recorded in the Swedish Hernia Register (SHR), their definitions and categorisation in this study

Postoperative complications occurring ≤ 30 days	Definition in the SHR	Category of complication
Recurrence	Recurrent hernia on the same side	
Urinary retention	Urinary retention requiring catheterisation	Urinary tract complications
Superficial hematoma	Bleeding from a minor vessel in the incision or dissection area, ventral of the transversalis fascia, including port incision	Hematomas
Deep hematoma	Bleeding from a major vessel intraabdominal or preperitoneal dorsal of the transversalis fascia	Hematomas
Superficial surgical site infection	Wound infection in the surgical incision or port incision	Surgical site infections
Deep surgical site infection/abscess	Infection dorsal of the transversalis fascia, preperitoneal or intraabdominal	Surgical site infections
Systemic infection	Sepsis	
Pain	Severe pain demanding prolonged pharmacological treatment, readmission, or reoperation	Acute postoperative pain
Intestinal obstruction	Intestinal obstruction	
Intestinal damage	Iatrogenic intestinal damage diagnosed postoperatively	
Urinary bladder damage	Iatrogenic bladder damage diagnosed postoperatively	
Testicular damage	Iatrogenic damage on vas deferens, testicular vessels, pain/atrophy of the testicle diagnosed postoperatively	
Cardiovascular event	Cardio or pulmonary event occurring postoperatively	
Seroma	Collection of fluid (blood, lymph) expanding the space of the pseudo hernia	Seromas
Death	Death occurring within 30 days postoperatively without any of above complications	

Postoperative complications occurring in the operated groin, and thus theoretically having some association with the risk for persisting pain, were considered, while systemic complications (e.g., cardiovascular event or systemic infection) were not investigated. Postoperatively diagnosed iatrogenic injury to the testis, bladder or intestine were not included in the analyses as these events were very rare. The postoperative complications registered were grouped into five categories: hematoma, surgical site infection, seroma, urinary tract complication and acute postoperative pain (Table 1).

Since the rates of complications vary according to hernia repair technique, the study cohort was divided into two groups based on the surgical approach: open anterior mesh repair and endo-laparoscopic repair with preperitoneal mesh placement.

Descriptive statistics and statistical analyses were performed using SPSS 22.0 (IBM Corp. 2013. IBM SPSS Statistics for windows, Version 22.0. Armonk, NY: IBM Corp.). Ordered logistic regression analyses were performed with self-reported pain at 1 year, according to the PROM seven-grade scale, as dependent variable. The postoperative complication categories (hematoma, surgical site infection, urinary tract complication, seroma, acute postoperative pain) were assessed as potential risk factors and entered as independent variables, along with age at surgery (years, continuous), sex (male/female), smoking habit (yes/no), and BMI (continuous, kg/m^2^). Separate analyses were performed for the two surgical method groups, open anterior mesh repair and endo-laparoscopic repair, respectively.

Non-responders were compared to the study group regarding demography, surgical technique, and reported postoperative complications using chi-square test for analysing categorical variables and *t* test for analysing continuous variables.

## Results

**Fig. 1:**
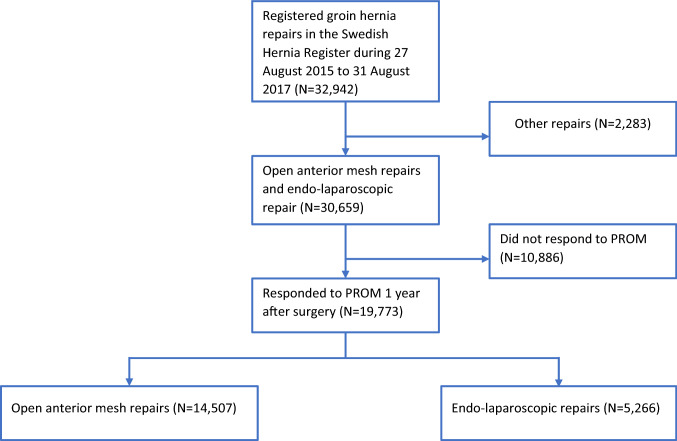
Flow chart showing inclusions, exclusions, responders and grouping

During the study period, 32,942 hernia repairs were registered in the SHR. After exclusion of 2283 cases not registered as open anterior mesh repair or endo-laparoscopic repair, 30,659 repairs remained eligible for inclusion. Altogether 19,773 responses to the PROM questionnaire were registered in the study group, while 10,886 did not respond, yielding a response rate of 64.5% (Fig. 1).

The study group (with responses to the PROM, *n* = 19,773) consisted of 91.4% males and 8.6% females. Mean age at surgery was 62.3 years. There were 4.1% smokers and mean BMI was 25.2 kg/m^2^. Hernia repair was performed with open anterior mesh repair in 73.4% cases and with endo-laparoscopic repair in 26.6% cases. Sex distribution in the two surgical method groups is presented in Table 2.

**Table 2:** Baseline data of responding patients operated during the study period, and registered in the Swedish Hernia Register, regarding age, gender, smoking, BMI and registered postoperative complications

Baseline data	Open anterior mesh repairs (*N* = 14,507)	Endo-laparoscopic mesh repairs (*N* = 5266)	All eligible responders (*N* = 19,773)
Mean age, years (standard deviation)	65.5 (12.9)	60.4 (14.3)	62.3 (13.5)
Sex			
Male	14,170 (97.7%)	3909 (74.2%)	18,079 (91.4%)
Female	337 (2.3%)	1357 (25.8%)	1694 (8.6%)
Smokers	602 (4.1%)	200 (3.8%)	802 (4.1%)
Mean BMI, kg/m^2^ (standard deviation)	25.3 (3.1)	24.9 (3.2)	25.2 (3.1)
Urinary tract complication	416 (2.9%)	321 (6.1%)	737 (3.7%)
Surgical site infection	258 (1.8%)	29 (0.6%)	287 (1.5%)
Hematoma	380 (2.6%)	62 (1.2%)	442 (2.2%)
Seroma	169 (1.2%)	55 (1.0%)	224 (1.1%)
Acute postoperative pain	180 (1.2%)	62 (1.2%)	242 (1.2%)

Definition and categorization of the registered postoperative complications are presented in Table 1. The registered complication rates for open anterior mesh repairs in the study group were 2.6% hematoma, 1.8% surgical site infection, 1.2% seroma, 2.9% urinary tract complication, and 1.2% acute post-operative pain. The corresponding rates for endo-laparoscopic repairs in the study group were 1.2% hematoma, 0.6% surgical site infection, 1.0% seroma, 6.1% urinary tract complication and 1.2% acute post-operative pain. Detailed distribution of complications is presented in Table 2.

In the groups of patients undergoing open anterior mesh repair (*n* = 20,735) or endo-laparoscopic repair (*n* = 9,924), response rates stratified for sex were 1,694/2,809 (60.3%) for females and 18,079/27,850 (64.9%, *p* < 0.001) for males. Mean age at surgery was 64.2 years (SD 13.5) in the study group and 58.0 years (SD 16.5) in the non-responder group (*p* < 0.001).

In the groups of patients undergoing open anterior mesh repair or laparoscopic repair the complication rates among responders and non-responders were as follows: 442/19,773 (2.2%) vs 258/10886 (2.4%) for hematoma (*p* = 0.450); 287/19,773 (1.5%) vs 169/10,886 (1.6%) for surgical site infection (*p* = 0.485); 224/19,773 (1.1%) vs 129/10,886 (1.2%) for seroma (*p* = 0.682); 737/19,773 (3.7%) vs 396/10,886 (3.6%) for urinary tract complication (*p* = 0.691); and 242/19,773 (1.2%) vs 243/10,886 (2.2%) for acute postoperative pain (*p* < 0.001).

**Fig. 2:**
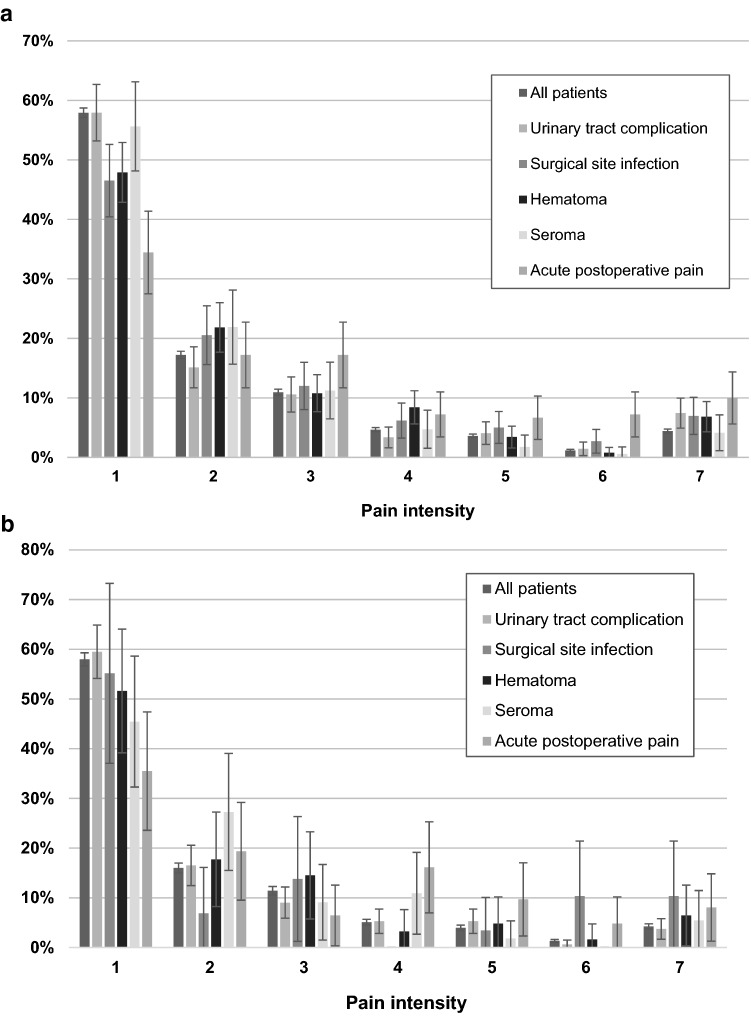
** a** Reported pain: 1-no pain; 2-pain can be ignored; 3-pain cannot be ignored, but it does not affect everyday activities; 4-pain cannot be ignored, and it affects everyday activities; 5-pain prevents most activities; 6-pain necessitates bed rest; and 7-pain requires immediate medical attention; and registered postoperative complications in patients having undergone open anterior mesh repair. **b** Reported pain: 1-no pain; 2-pain can be ignored; 3-pain cannot be ignored, but it does not affect everyday activities; 4-pain cannot be ignored, and it affects everyday activities; 5-pain prevents most activities; 6-pain necessitates bed rest; and 7-pain requires immediate medical attention; and registered postoperative complications in patients having undergone endo-laparoscopic mesh repair

The distribution of self-reported grades of persisting pain is shown as bar charts according to open anterior repair (Fig. 2a) or endo-laparoscopic repair (Fig. 2b). The distribution of self-reported pain grades according to the seven-grade scale 1 year after surgery in the open anterior mesh repair group was: 8402 grade 1(57.9%), 2498 grade 2 (17.2%), 1589 grade 3 (11.0%), 677 grade 4 (4.7%), 526 grade 5 (3.6%), 173 grade 6 (1.2%), and 642 grade 7 (4.4%).

The distribution of self-reported pain grades in the endo-laparoscopic repair group was: 3052 grade 1 (58.0%), 843 grade 2 (16.0%), 602 grade 3 (11.4%), 268 grade 4 (5.1%), 209 grade 5 (4.0%), 69 grade 6 (1.3%), and 223 grade 7 (4.2%).

Uni- and multivariable ordered log-odds regression analyses in the open anterior mesh repair group showed that hematoma, surgical site infection and acute postoperative pain were associated with CPIP 1 year after hernia repair, while urinary tract complication and seroma were not. Age was inversely related to persistent pain; the odds ratio for CPIP decreased for every 1-year increase in age at the time of surgery. BMI was associated with persistent pain; the odds ratio for CPIP increased for every step increase in BMI. Females, who had an open anterior repair, also had an increased odds ratio for developing CPIP (Table 3).

**Table 3:** Risk factors for Chronic Postoperative Inguinal Pain (CPIP) one year after surgery in (*n* = 14,507) patients with open anterior mesh repair. Univariable and multivariable ordinal regression analysis

	Univariable analysis		Multivariable analysis	
	Ordered regression odds ratio (95% confidence interval)	*P*	Ordered regression odds ratio (95% confidence interval)	*p*
Age (years)	0.97 (0.964–0.975)	< 0.001	0.97 (0.964–0.975)	< 0.001
Female	2.08 (1.306–3.304)	0.002	2.60 (1.596–4.227)	< 0.001
Smoker	1.73 (1.212–2.460)	0.002	1.52 (1.059–2.178)	0.023
BMI (kg/m^2^)	1.09 (1.062–1.114)	< 0.001	1.07 (1.047–1.099)	< 0.001
Urinary tract complication	1.16 (0.751–1.780)	0.508	1.50 (0.953–2.350)	0.079
Surgical site infection	2.90 (1.722–4.887)	< 0.001	2.18 (1.271–3.733)	< 0.001
Hematoma	2.37 (1.531–3.656)	< 0.001	2.03 (1.300–3.184)	< 0.001
Seroma	1.03 (0.524–2.023)	0.932	0.88 (0.435–1.762)	0.707
Acute post-operative pain	10.84 (5.929–19.815)	< 0.001	7.46 (4.018–13.868)	< 0.001

In the endo-laparoscopic repair group, acute postoperative pain was associated with CPIP 1 year after surgery, while the other postoperative complications were not. Older age was associated with a decreased odds ratio for CPIP, while females, smokers, and patients with a higher BMI had increased odds ratios for persistent pain (Table 4).

**Table 4:** Risk factors for Chronic Inguinal Postoperative Pain (CPIP) one year after surgery in (*n* = 5266) patients with endo-laparoscopic mesh repair. Univariable and multivariable ordinal regression analysis

	Univariable analysis		Multivariable analysis	
	Ordered regression odds ratio (95% confidence interval)	P	Ordered regression odds ratio (95% confidence interval)	p
Age (years)	0.98 (0.975–0.991)	< 0.001	0.98 (0.973–0.989)	< 0.001
Female	1.69 (1.288–2.218)	< 0.001	2.13 (1.603–2.844)	< 0.001
Smoker	4.35 (2.404–7.852)	< 0.001	4.84 (2.655–8.810)	< 0.001
BMI (units)	1.13 (1.081–1.167)	< 0.001	1.15 (1.102–1.191)	< 0.001
Urinary tract complication	0.85 (0.512–1.416)	0.537	1.10 (0.652–1.866)	0.715
Surgical site infection	2.47 (0.524–11.695)	0.253	2.33 (1.478–11.402)	0.296
Hematoma	1.77 (0.600–5.224)	0.301	1.85 (0.614–5.585)	0.275
Seroma	2.18 (0.700–6.808)	0.179	2.40 (0.746–7.727)	0.141
Acute post-operative pain	9.75 (3.483–27.290)	< 0.001	9.35 (3.184–27.479)	< 0.001

## Discussion

In this large population-based cohort study, we found an increased risk for CPIP among patients suffering from acute post-operative pain after groin hernia repair. With open anterior mesh repair the risk for CPIP was also increased in patients with postoperative hematoma or surgical site infection. There was an apparent difference in patient characteristics between the group who had endo-laparoscopic repair, who were younger and more often female, and the group who had open repair.

Acute postoperative pain was a strong risk factor for CPIP after open anterior as well as after endo-laparoscopic repair. The definition of acute postoperative pain in the SHR is “severe pain resulting in prolonged pharmacologic treatment, readmission or reoperation” [16]. Acute postoperative pain has been shown to be more frequent after open anterior repair than after endo-laparoscopic repair [20–23]. This can be explained by the surgical procedure during an open hernia repair, with a wider incision and directly interfering with the main inguinal sensory nerves.

In a previous study we showed that patient-reported “early severe postoperative pain in the groin” was associated with “chronic groin pain” 8 years after open hernia repair [14]. The present study demonstrated that the registered postoperative complication “acute postoperative pain” increases the risk for CPIP after both open anterior mesh repair and endo-laparoscopic repair.

Several habitual risk factors for acute postoperative pain are known including history of chronic pain, preoperative groin pain, and younger age [3, 8, 24]. An association between acute and persisting postoperative pain has been shown in previous studies [12, 13]. Adequate management of severe early postoperative pain is important to avoid the conversion to CPIP. The results of this large cohort study reveal that acute postoperative pain is associated with CPIP. This finding underlines the importance of avoiding all possible causes of acute postoperative pain during the surgical procedure, such as minimal skin incision, atraumatic surgical dissection, and careful nerve handling. Preoperative nerve blockade and generous use of local anaesthesia have important roles to play in this respect.

To minimise the risk for acute postoperative pain, previous history should be carefully considered before planning for surgery. The use of a less traumatic surgical method, such as endo-laparoscopic repair has been reported to minimise the risk for postoperative complications [23]. In cases where surgery is indicated despite a history of factors that increase the risk for persisting pain, the procedure should preferably be performed by an experienced surgeon.

Surgical site infection was a significant risk factor for persistent pain after open anterior mesh repair, but not after endo-laparoscopic repair. This category of complications includes superficial infection and deep infection. Open anterior mesh repair is associated with a higher rate of surgical site infection compared to endo-laparoscopic repair [25], and this was also the case in this study. Postoperative wound infection can delay the healing process as well as cause scarring, potentially resulting in nerve damage, which may increase the risk for CPIP.

Perioperative antibiotic prophylaxis is seldom used today as part of the campaign to reduce the prevalence of resistant pathogens [26]. Prophylactic use of antibiotics should, however, be considered in cases, where there is an increased risk for postoperative surgical site infection, such as patients with immunosuppression, diabetes, and obesity [27–29]. Distinct patient information and early postoperative follow-up could help to diagnose postoperative surgical site infection requiring treatment, at an early stage.

The hematoma category in this study included both deep and superficial hematoma. Hematoma was associated with CPIP after open anterior mesh repair, but not after endo-laparoscopic repair. The risk for hematoma is generally higher after open anterior mesh repair than after endo-laparoscopic repair [23]. Intraoperative and postoperative bleeding causing hematoma may be associated with surgical technique, and therefore, the surgical performance could be a confounder causing both CPIP and wound complications. Ungentle surgical technique may be seen in acute situations, with less experienced surgeons and in patients with complicating factors, such as recurrent hernia or obesity [30, 31]. Patient-related risk factors for postoperative hematomas such as antithrombotic medication or the use of omega-3 fatty acids should also be noted and preferably paused prior to surgery [32–34]. Preoperative planning and optimising of the surgical technique are the cornerstones of postoperative hematoma risk reduction.

Local surgical wound complications, such as surgical site infection and hematoma in the surgical area were associated with CPIP after open anterior mesh repair in this study, even though the complication rates were low.

One hypothesis, not possible to evaluate from the present material, is that the increased odds ratios presented may be a result from confounding, where, for example, traumatic dissection causes local postoperative complications as well as persisting postoperative pain. The extent of tissue trauma inflicted by the surgical technique is not registered in the SHR, but the potential association between traumatic technique, postoperative complications and persisting pain gives indirect support for recommending careful technique to reduce the risk of persisting pain.

The results of this study are statistically significant but since the analyses are based on relatively rare events in a large cohort, the clinical importance must be evaluated with this in mind. The absolute risk for one of these events occurring is low and accordingly a moderate increase in the incidence of a specific complication will have limited impact on the rate of CPIP. Nevertheless, this study provides detailed information regarding the association between specific postoperative complications and CPIP as well as confirming findings from previous smaller studies.

The postoperative complication categories urinary tract complication and seroma were not significant risk factors for CPIP regardless of technique used. In contrast to hematoma, surgical site infection and acute postoperative pain, urinary tract complications are not obviously associated with a surgical dissection technique causing tissue damage and nerve injuries. This could explain the lack of association with CPIP. Postoperative seroma may be associated with surgical technique but there might be predisposing factors which are not associated with surgical technique, such as coagulopathy, congestive liver disease and cardiac insufficiency [35].

Younger age and female sex are well-established predictors of CPIP and were independent risk factors in this study too [3, 8, 12]. BMI was another known risk factor that showed to be a significant predictor for CPIP. Obesity often causes difficult conditions during dissection, which could explain this finding [31]. Smoking habit was a risk factor for chronic pain after endo-laparoscopic repair but not after open anterior mesh repair. Smoking habit was a risk factor for chronic pain after endo-laparoscopic repair as well as after open anterior mesh repair. Smoking impairs wound healing, in addition to other possible effects of smoking on risk for chronic pain.

CPIP is one of the most significant long-term complications after inguinal hernia repair. It must be considered when planning and optimizing groin hernia surgery. In patients with an asymptomatic hernia, the risk for intestinal strangulation should be weighed against the risk for CPIP. In some cases, the strategy “watchful waiting” could be preferred [36–39].

The main strength of this study is the large population of almost 20,000 individuals. The Swedish national register with its high coverage and verified validity, further underlines the strength of the study, and increases its external validity. Use of the PROM questionnaire for reporting pain 1 year after surgery combined with data on specific postoperative complications in the SHR provide a deeper understanding of the association between postoperative complications and CPIP that previous smaller studies have suggested (5, 14).

The study also has limitations. Preoperative pain could not be included as an independent variable in the analyses of this study, even though it is a known predisposing factor for chronic pain, since that information is presently not included in the SHR. The response rate was 64.5% which was lower than desired. Since the PROM is sent to all hernia patients rather than to a selection of patients who have accepted inclusion in a study involving a specific follow-up regime, 64.5% may be considered reasonable and may not even be lower than expected. Furthermore, this study was not designed as a controlled study. The non-responder group was investigated to examine any selection bias. It differed from the responder group in distribution of method of repair, age, and sex, although the differences were not large, and it is possible that the postoperative outcome regarding pain between non-responders and responders differed. On the other hand, the non-responder group did not differ substantially from the responder group regarding reported postoperative complications in the register. Together, these facts suggest that the results of the present study are applicable to a large population.

## Conclusion

Acute postoperative pain was a strong predictor for CPIP 1 year after surgery after both open anterior mesh repair and endo-laparoscopic repair. Hematoma and surgical site infections also moderately increased the risk for persisting groin pain after open anterior mesh repair. Considering the moderate influence and the relatively low rates of these specific complications, their role in the pathogenesis of CPIP is probably limited. Our findings suggest that other strategies are required to reduce the problem of CPIP, in particular improvement of surgical technique during inguinal hernia surgery.

## Data Availability

All data is stored at the institution and is available for reviews.
