# Correction to: Do postoperative complications correlate to chronic pain following inguinal hernia repair? A prospective cohort study from the Swedish Hernia Register

**DOI:** 10.1007/s10029-023-02743-w

**Published:** 2023-01-16

**Authors:** A. Olsson, G. Sandblom, U. Franneby, A. Sondén, U. Gunnarsson, U. Dahlstrand

**Affiliations:** 1 Department of Clinical Science and Education Södersjukhuset, Karolinska Institutet, Stockholm, Sweden; 2 Department of Clinical Science, Intervention and Technology, Karolinska Institutet, Stockholm, Sweden; 3 Department of Surgical and Perioperative Sciences, Surgery, Umeå University, Umeå, Sweden; 4 Department of Surgery, Södersjukhuset, Stockholm, Sweden

**Correction to: Hernia** 10.1007/s10029-021-02545-y

The statistical analyses showed that smoking was a significant risk factor for CPIP after* both* open anterior mesh repair and after endo-laparoscopic repair, which is correct reported in Table 3 and Table 4.

In the Discussion section however, smoking habit is discussed as “a risk factor for chronic pain after endo-laparoscopic repair* but not* after open anterior mesh repair.” in our published paper.

The original article has been corrected.

